# Radiographic Analysis of the Beef Cattle Digits Slaughtered after 114 Days of Confinement

**DOI:** 10.1155/2024/5512555

**Published:** 2024-06-28

**Authors:** Wanessa Patrícia Rodrigues da Silva, Paulo José Bastos Queiroz, Paulo Henrique Jorge da Cunha, Antônio Dionísio Feitosa Noronha Filho, Emmanuel Arnhold, Juliano José de Resende Fernandes, Kaique de Souza Nascimento, Naida Cristina Borges

**Affiliations:** School of Veterinary Medicine and Animal Science Federal University of Goiás, Goiânia-Nova Veneza Highway, Kilometer 8, Samambaia Campus, Goiânia 74690-900, Goiás, Brazil

## Abstract

Foot disorders are responsible for up to 5% of confinement losses. Identifying the cause of lameness and applying the correct treatment are crucial measures. The possibility of determining the probable origins of digital lesions, their extension, and assistance in cases in which it was not possible to reach a definitive clinical diagnosis demonstrates that the radiographic evaluation provides information of great importance on cattle digits. Thus, this study aimed to investigate possible radiographic changes in the hoof, bone structures, and soft tissues of the digits of Nelore bulls at the end of the confinement period. Regarding the main signs, 100% (*n* = 24) showed proliferation of enthesophytes and osteophytes and irregular contours, 62.5% (*n* = 15) enlargement of vascular channels, 37.5% (*n* = 9) osteolysis, 33.4% (*n* = 8) gas content in the white line region, 20.84% (*n* = 5) gas content in the dorsal lamina region, and 4.16% (*n* = 1) presented palisade periosteal reaction. Ten (41.7%) out of 24 (100%) animals evaluated at the end of the confinement presented lameness, three animals of score three, four animals of score two, and three animals of score one. The higher the score indicating the severity of the signs observed in the visual assessment of the radiographs, the higher the internal angle values (moderate correlation of 0.5 and *p* < 0.05). A higher prevalence of osteo-proliferative radiographic changes was identified at the end of the 114-day confinement period in the third phalanx of Nellore cattle even in animals that had no lameness.

## 1. Introduction

Intensive management favors the occurrence of inflammatory or infectious foot disorders in cattle. Several factors, such as management [1, 2], nutrition [3], facilities, and the type of floor where the animals are kept can cause morphological changes in the hoof, which influences the distribution of forces on the hoof support surface [2].

Lameness clinical manifestation in beef cattle can generate serious economic consequences for producers. Foot disorders are responsible for up to 5% of confinement losses [4]. In addition, lameness is associated with an unsatisfactory result of reproductive health in beef breeding bulls [5].

Identifying the cause as soon as possible and applying the correct treatment is crucial, as its origin is multifactorial [4]. Foot diseases in beef cattle have been underestimated in several regions of Brazil and the world [1, 6], which characterizes an important barrier for the detection and treatment of lameness [6].

The absence of an early diagnosis in the field may contribute to increase the severity and diversification of foot disorders [1]. Inadequate control and prophylaxis measures relative to foot disorders demonstrate the importance of establishing adequate programs for preventing lameness in beef cattle [1, 5, 6].

The adoption of radiographic examination for orthopedic evaluation in the clinical routine of cattle farming has been growing over the years. Radiographic examination in dairy cows has been widely used in the assessment of digits, helping in early diagnosis, determining prognosis, and directing effective treatments [7–9]. The possibility of determining the probable origins of digital lesions, as well as determining their extent and helping in cases whose definitive clinical diagnoses could not be reached, demonstrates that radiographic evaluation provides information of great importance on cattle digits [7, 10–13]. Approximately 30% of bulls that did not present lameness had radiographic changes and the digits were the most affected regions, with enthesopathy, septic arthritis, fractures, and degenerative joint disease among the most frequent radiographic changes [13].

A study carried out by Gantke et al. [10], including beef cattle breeds, identified several radiographic changes related to laminitis and its sequelae. However, the misalignment of the third phalanx could not be objectively verified due to the absence of radiographic measurements in normal hooves. Subsequently, Hage et al. [12] determined the radiographic pattern of normality of measurements and angles in digits of Nellore females.

This study aimed to investigate possible radiographic changes in the hoof, bone structures, and soft tissues of the digits of Nellore cattle at the end of the confinement period.

## 2. Materials and Methods

### 2.1. Animals and Management

After confinement, 24 intact male Nellore cattle with an average age of 22 months were sent for slaughter in a slaughterhouse and the collection of thoracic and pelvic digits was authorized. The project was previously approved by the Ethics Committee on the Use of Animals under protocol number 111/19 and carried out at the Experimental Confinement of Beef Cattle of the School of Veterinary and Animal Science of the Federal University of Goiás.

The animals were confined for 114 days from July to October (dry period with the transition to the rainy period at the beginning of October) and allocated to three collective stalls (7.7 × 10 m), with eight animals being distributed per stall. The animals had free access to water in 500 liter drinking troughs shared between two stalls and were fed with feed containing fresh sugarcane bagasse (11.18%), finely ground corn (66.83%), soybean hulls (9.35%), proteinaceous corn meal (5.70%), soybean meal (3.34%), and mineral core (2.63%).

Lameness score was determined in the last week of the confinement, close to sending the animals to the slaughterhouse. The pieces were collected from the carpometacarpal and tarsometatarsal disarticulation and stored in a freezer at 20°C.

The thoracic (*n* = 96) and pelvic (*n* = 96) limbs were thawed and sanitized with water, brush, and soap for further analysis of the digits and the hoof capsule. The radiographic examination was performed after they were dry.

### 2.2. Lameness Score

A locomotion scoring scale was adopted for lameness assessment with scores between 0 and 3 [14] (Table 1). The evaluation was performed by one of the authors, who has experience in this evaluation. The animals were led individually in a corridor beside the stalls with an earthen floor, without irregularities.

### 2.3. Radiographic Examination

The bony structures, hoof, and soft tissue topography of the third phalanx were examined using radiographs in the lateromedial, mediolateral, and palmar/plantarodorsal projections. The examinations were performed using bags filled with sand for supporting and fixing the digits and a Philips fixed radiographic, computed radiography (CR), device model KL.74/20.40 (Philips Heathcare®, Biassono, Italy) with a capacity of 500 mA and 125 kVp, with the technique of 75 kVp and 30 mAs.

The radiographic images were evaluated and interpreted by a single experienced radiologist, without knowledge of the lameness score information. The images were evaluated using the free version of the Horos software (version 3—LGPL3.0) and interpreted for the presence of the following parameters [7, 10, 15]: osteophytes, bone lysis, bone remodeling, enthesophytes, cysts, and spiculated periosteal reaction.

Radiographic changes were classified into scores according to lesion characteristics, location, and severity (Table 2).

**Figure 1:**
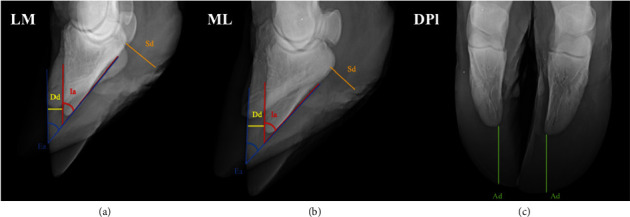
Radiographs of the third phalanx of the right pelvic limb of a Nelore bull in lateromedial (a), mediolateral (b), and plantarodorsal (c) exhibitions. Distance between the dorsodistal edge of the third phalanx and the dorsal wall of the hoof (Dd), distance between the solar edge of the flexor tubercle and the hoof (Sd), external angle over the silhouette of the dorsal edge of the hoof and the sole of the third phalanx (Ea), internal angle on the silhouette of the third phalanx (Ia), and distances between the apex of the hoof and the apex of the third phalanx in plantarodorsal (Ad).

Measurements were performed still using the radiographic images to verify the positioning of the third phalanx (3P) relative to the hoof capsule, as shown in the diagram of Figure 1. The measurements consisted of the distance between the dorsal face of the 3P and the hoof wall [8, 10], plantar/palmar distance of the 3P and solar face of the hoof [7, 15], and angulation of the third phalanx relative to the hoof and dorsal wall of the 3P with the starting point on the solar face of the flexor tubercle [7, 12].

In addition, measurements of the distances between the apex of the third phalanx to the apex of the hoof capsule in the plantaro/palmarodorsal projections of the medial and lateral digits of pelvic and thoracic limbs were proposed by the researchers of this study to estimate the hoof growth relative to the distal phalanges.

### 2.4. Statistical Analysis

Descriptive results related to radiographic examinations of lameness were presented as a frequency [16]. Lameness scores and objective and subjective radiographic evaluation were assessed to determine the existence of a correlation between them and statistical difference (Table 3) through analysis of variance (ANOVA), test of means, Scott–Knott (parametric data) and Kruskal–Wallis nonparametric data, and Spearman correlation [16–18].

## 3. Results

Table 4 shows the individual and the mean values of measurements from the apex to hoof in palmaro/plantarodorsal projections and dorsal and solar distances, and internal and external angles.

The means shown in Table 4 were grouped and tested for lameness scores (Table 3). The animals that presented a score of 3 for lameness had higher values in the measurements of dorsal distances and internal angles and lower values of external angles in pelvic and thoracic limbs.

**Figure 2:**
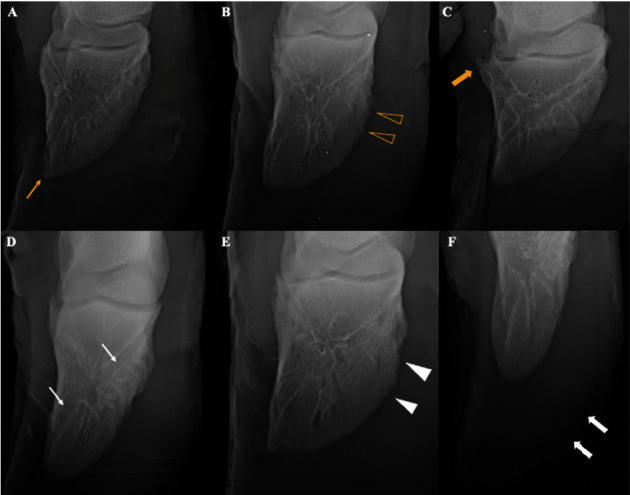
Radiographs in plantarodorsal and palmarodorsal exhibitions, demonstrating the main radiographic signs observed in the third phalanx of Nellore cattle after 114 days of confinement: (A) osteophytes at the apex of the third phalanx (yellow arrow), (B) spiculated periosteal reaction (empty arrowhead), (C) axial enthesophyte (thick yellow arrow), (D) widening of vascular channels (white arrows), (E) irregular contours in the abaxial face and apex (full arrowhead), and (F) gas in the white line region (thick white arrows).

The radiographic evaluation showed that all animals had signs, the most prevalent being osteo-proliferative signs (Figure 2). Regarding the main signs, 100% (*n* = 24) showed proliferation of enthesophytes and osteophytes and irregular contours, 62.5% (*n* = 15) enlargement of vascular channels, 37.5% (*n* = 9) osteolysis, 33.4% (*n* = 8) gas content in the white line region, 20.84% (*n* = 5) gas content in the dorsal lamina region, and 4.16% (*n* = 1) presented palisade periosteal reaction.

Ten (41.7%) out of the 24 (100%) animals evaluated at the end of the confinement period had lameness, with scores of three (*n* = 3), two (*n* = 4), and one (*n* = 3) (Chart 4). A weak correlation with no statistical significance was observed between lameness and subjective radiographic evaluation.  Table 5 shows the details of the signs and the classification of lameness and radiographic scores. Other 14 (58.3%) animals did not present lameness, but bone remodeling signs were observed in the third phalanx, with radiographic scores similar to those observed in animals with lameness scores from 1 to 3.

The classification of radiographic scores showed that eight animals (33.4%) presented a score of 3, six (25%) animals had a score of 4, two (8.4%) animals presented a score of 2, seven (29.16%) animals had a score of 6, and one (4.16%) animal had a score of 5.

The correlation coefficients showed that the higher the score indicating the severity of the signs observed in the visual evaluation of the radiographs (proliferative and lytic, bone fissures, gas content in dorsal laminae, and spiculated periosteal reaction), the higher the values of the internal angles (moderate correlation of 0.5 and *p* < 0.05).

The evaluation of the relationship between the measurements on the radiographic examination showed that longer apical distances in pelvic and thoracic limbs influenced longer dorsal distances (strong correlation of 0.7 and *p* < 0.05). The measurements of angles showed a strong correlation (0.7 and *p* < 0.05) between the internal and external angles in the pelvic and thoracic limbs.

EOBF and JSC: Enthesophyte and osteophyte bone formation with a jagged surface contour.

The comparison between angles and distances showed that both the internal and external angles decreased as the apical distance increased in pelvic and thoracic limbs (strong negative correlation of −0.7 and *p* < 0.05). Only the external angle in pelvic and thoracic limbs presented a moderate negative correlation (−0.5 and *p* < 0.05) with the dorsal distance, indicating that the smaller the external angle, the longer the dorsal distance.

## 4. Discussion

The identification of higher values in measurements of dorsal distances and internal angles and lower values of external angles in pelvic and thoracic limbs in animals with a score of 3 may be associated with pain, a factor that biomechanically alters or prevents normal movement and causes new injuries [19]. It may be associated with the loss of mechanical stability of the third phalanx, which interferes with the structures of the suspensory apparatus, making it insufficient to absorb the impact of the third phalanx on the corium. The third phalanx without adequate suspension and damping changes its position within the restricted space of the hoof capsule with all the weight supported by the limb [20].

The fact that more than half of the evaluated animals presented no lameness detected by the locomotion score but had bone remodeling signs in the third phalanx can be explained by the chronicity of the lesions, as lameness may not be evident in cases of chronic laminitis, which presents hoof deformations. The disease may progress silently for a long-term asymptomatic subclinical period [20]. These findings corroborate the information obtained by Barbosa et al. [21], who observed that the evaluated cattle presented no lameness even with radiographic signs and chronic lesions may not promote nociceptive signs in cattle locomotion.

Thus, the severity of radiographic findings in the third phalanx may not be related to the locomotion score. Hoof lesions may return after some period, although corrective hoof trimming is performed on affected digits, leading the animal to lameness resolution or reduction [22]. This information reinforces the importance of using radiographic examination to detect digital signs, which can evolve, worsen, and later affect the distal interphalangeal joint.

The establishment of the score indicating the severity of radiographic signs by the researchers of this study confirms the one developed by Nouri et al. [22], who considered the presence of bone neoformation, osteitis, and the presence of gas content, three factors considered to assess the disease severity. In addition, the presence of bone lysis was considered a worsening factor in this study.

The directly proportional relationship between higher values of the radiographic score and higher values of internal angles can be explained by bone remodeling, observed with significant differences between the lateral surface of the proximal and distal parts with the other surfaces [22]. Remodeling of the third phalanx can occur as a result of bone necrosis by compromising the regional blood supply, affecting nutrition and oxygenation [8, 20].

The measures of internal and external angles decrease as the hoof elongates apically, which can be explained by the sinking of the third phalanx. The cause of the 3P sinking can be attributed to damage to the chorion [8, 23], which is further potentiated by this sinking, related to laminitis [19, 24, 25]. Bone development in subclinical laminitis occurs mainly in an abaxial-axial direction, which refers to the narrowing of the space between the solar surface of the distal phalanx and the internal surface of the hoof capsule, which may lead to irregularity of the solar surface [19, 26, 27]. The flexor tubercle is the most important site on the solar surface for these alterations [19], which also supports the decision of the authors of this study to perform only one measurement on the solar surface with the flexor tubercle as the point of origin and the measurement in the apical region.

The permanence in a confined environment and on an earthen floor may be one of the factors associated with the increase in the apical distance, as a thicker hoof wall was observed in confined beef cattle, causing a change in their external structure [2, 24, 25].

The prevalence of osteo-proliferative radiographic signs observed in all animals is associated with lesions related to chronic laminitis, corroborating the findings of higher prevalence in other studies [7, 8, 21, 23, 24]. In addition, Nguhiu-Mwangi and Mbithi [23] mentioned dilatation of vascular channels and osteolysis, the latter not very prevalent in this study [28–30].

## 5. Conclusions

In the third phalanx of the Nellore cattle, without lameness, osteo-proliferative radiographic signs were identified at the end of the 114-day confinement period. The establishment of the radiographic lesion severity score, proposed in this study, may become a facilitating tool for the application of field evaluations by buiatricians.

It is important to emphasize that these are experimental data, which can provide relevant information for the diagnosis of foot diseases in cattle, and require application in live animals.

## Figures and Tables

**Table 1:** Lameness score ranging from 0 to 3 in beef cattle.

Score	Designation	Description
0	Normal	The animal walks normally, with no apparent lameness or change in gait. The pelvic limbs land in a similar location to the thoracic limbs, with the back level
1	Mild lameness	The animal presents short strides when walking, lowering its head slightly. The animal does not limp when walking. There may be slight arching of the back when walking
2	Moderate lameness	The animal has evident flaccidity, favoring the affected limb(s) that still support the weight. A slight nod of the head is present when the animal walks. It may have back arching
3	Marked lameness	The animal applies little or no weight to the affected limb and is reluctant or unable to move. The back will arch during the walk, with the head swaying and lameness detected. It may have an arched back when standing up and walking

Source: Zimpro [14].

**Table 2:** Score for evaluation of radiographic changes in the digits of Nellore cattle after a 114-day confinement period.

Score	Description
0	No changes
1	Proliferative signs (enthesophytes, osteophytes, and irregular contours)
2	Proliferative signs with bone remodeling and channel widening
3	Proliferative signs with bone remodeling, channel widening, and gas content in the white line, and lateroapical to the third phalanx
4	Proliferative and lytic signs, bone remodeling, and channel widening
5	Proliferative and lytic signs, bone remodeling, channel widening, and gas content in the white line
6	Proliferative and lytic signs, bone fissures, gas content in dorsal laminae, and spiculated periosteal reaction

**Table 3:** Parametric and nonparametric data with their respective medians and mean and tests of means.

Variable	Claudication score				p-ANOVA	p-Shapiro–Wilk	p-Kruskal–Wallis
	0	1	2	3			
Radiographic score	4	4	3.5	3	—	—	0.9036
TLDD	1.29^b^	1.14^b^	1.25^b^	1.78^a^	0.0061	0.1502	—
TLSD	2.4	1.96	2.48	2.34	0.0854	0.0782	—
TLAD	3.53^b^	2.98^b^	2.97^b^	5.16^a^	0.0356	0.9987	—
TLIA	49.15°	48,8°	50.42°	47.8°	0.6977	0.715	—
TLEA	49°^a^	49.1°^a^	50°^a^	43°^b^	0.0474	0.9853	—
PLDD	1.39^b^	1.4^b^	1.32^b^	1.9^a^	0.0261	0.065	—
PLSD	2,26	2.15	2.39	2.32	0.8423	0.084	—
PLAD	3.1^b^	2.3^b^	3.16^b^	4.81^a^	0.0357	0.125	—
PLIA	48.7°	49.8°	50.9°	48.01°	0.6838	0.2743	—
PLEA	47.9°^a^	49.8°^a^	51.5°^a^	39.8°^b^	0.0498	0.1183	—

Means followed by the same letter (a or b) do not differ from each other at a 5% significance.

**Table 4:** Means (Me) and standard deviations (SDs) of measurements of apical and dorsal distances (cm), internal and external angles (°) of thoracic (TL) and pelvic limbs (PL) of the lateral (L), and medial digits (M) of adult Nellore bulls after 114 days of confinement.

Animals	Apical distance				Sole distance				Dorsal distance				External angle				Internal angle			
	TL		PL		TL		PL		TL		PL		TL		PL		TL		PL	
	L	M	L	M	L	M	L	M	L	M	L	M	L	M	L	M	L	M	L	M
1	3, 0	2, 7	2, 7	2, 5	0, 8	2, 0	1, 8	2, 5	1, 1	2, 2	1, 3	2, 1	55, 8	58, 9	57, 4	52, 8	52, 5	54, 0	53, 2	52, 8
2	3, 5	3, 8	3, 3	3, 4	2, 5	2, 4	2, 4	2, 7	1, 1	2, 4	1, 2	2, 6	45, 9	45, 4	47, 5	46, 8	46, 7	46, 2	48, 6	47, 4
3	2, 9	2, 9	2, 6	2, 5	2, 5	2, 5	2, 3	2, 5	1, 2	2, 5	1, 4	2, 4	50, 4	51, 0	56, 1	47, 0	49, 2	51, 3	56, 2	46, 2
4	2, 3	2, 6	1, 7	1, 9	2, 1	1, 7	2, 2	2, 0	1, 1	1, 9	1, 4	2, 1	49, 6	51, 1	53, 9	52, 1	49, 7	50, 9	51, 7	51, 3
5	3, 1	3, 1	1, 8	1, 5	2, 4	2, 4	1, 9	1, 9	1, 2	2, 4	1, 2	1, 9	52, 4	45, 8	47, 7	52, 5	53, 1	46, 8	48, 2	51, 2
6	2, 2	2, 2	2, 4	1, 9	2, 3	2, 5	2, 3	2, 0	1, 0	2, 4	1, 3	2, 2	54, 0	49, 9	47, 4	51, 7	52, 8	47, 5	47, 5	51, 3
7	4, 3	4, 5	4, 1	4, 3	2, 3	2, 4	2, 0	2, 5	1, 5	2, 3	1, 7	2, 3	49, 3	45, 4	51, 1	47, 7	48, 4	45, 3	50, 5	49, 6
8	4, 2	4, 1	4, 2	4, 1	2, 2	2, 2	2, 1	2, 3	1, 2	2, 2	1, 2	2, 2	46, 9	48, 7	49, 8	50, 3	47, 9	47, 3	51, 3	51, 1
9	3, 3	3, 3	2, 8	2, 9	2, 3	2, 5	2, 4	2, 3	1, 1	2, 4	1, 2	2, 3	46, 5	48, 0	46, 9	50, 7	49, 7	48, 6	48, 9	51, 0
10	4, 8	4, 8	4, 7	4, 6	2, 0	2, 6	2, 1	2, 3	1, 5	2, 3	1, 5	2, 2	39, 5	40, 5	41, 6	23, 1	41, 9	42, 5	41, 5	40, 2
11	2, 3	2, 4	2, 2	1, 8	2, 1	2, 6	2, 0	2, 1	1, 1	2, 4	1, 2	2, 1	53, 2	50, 4	47, 4	48, 8	51, 1	51, 2	46, 0	48, 2
12	3, 9	4, 0	3, 9	3, 9	2, 3	2, 1	1, 7	1, 9	1, 6	2, 2	1, 7	1, 8	46, 3	46, 8	47, 6	48, 9	47, 0	47, 2	46, 6	45, 0
13	3, 0	3, 2	2, 9	3, 1	2, 0	2, 3	2, 2	2, 2	1, 2	2, 2	1, 3	2, 2	49, 8	47, 3	48, 0	43, 2	50, 5	46, 8	47, 9	45, 9
14	4, 3	4, 1	4, 0	4, 1	2, 3	2, 1	2, 0	2, 3	1, 6	2, 2	1, 6	2, 1	50, 1	48, 7	49, 9	48, 5	50, 6	47, 5	52, 7	46, 5
15	6, 5	6, 1	5, 5	5, 7	2, 6	2, 3	2, 6	2, 5	2, 3	2, 4	2, 4	2, 5	40, 1	43, 2	42, 4	33, 3	53, 1	55, 6	54, 4	52, 0
16	3, 4	2, 9	2, 2	2, 4	2, 4	2, 4	2, 0	2, 0	1, 4	2, 4	1, 4	2, 0	48, 7	49, 9	49, 7	54, 8	51, 5	49, 3	49, 5	54, 8
17	2, 5	2, 6	2, 5	2, 0	2, 6	2, 7	2, 2	2, 5	1, 1	2, 7	1, 2	2, 3	56, 0	54, 4	52, 7	53, 2	54, 1	54, 8	50, 8	53, 9
18	3, 6	4, 0	2, 8	2, 2	2, 6	2, 7	2, 2	2, 3	1, 2	2, 6	1, 2	2, 3	47, 5	52, 9	50, 3	51, 3	46, 6	54, 0	49, 3	52, 2
19	5, 1	4, 8	5, 3	4, 7	3, 1	3, 1	3, 2	3, 3	1, 5	3, 1	1, 5	3, 2	39, 3	46, 3	43, 4	49, 6	47, 0	47, 2	44, 0	45, 1
20	5, 0	4, 7	4, 1	4, 6	2, 3	2, 3	3, 0	2, 9	1, 5	2, 3	1, 8	2, 9	50, 4	47, 9	41, 9	42, 6	47, 0	47, 5	47, 3	46, 2
21	3, 7	3, 8	3, 3	3, 4	1, 9	1, 9	2, 2	1, 9	1, 4	1, 9	1, 3	2, 1	46, 6	44, 9	46, 4	44, 7	49, 3	47, 8	46, 0	43, 8
22	5, 0	4, 9	4, 9	4, 7	2, 9	3, 2	3, 4	2, 4	1, 4	3, 0	1, 8	2, 9	44, 4	43, 8	33, 1	33, 4	44, 3	46, 0	42, 8	43, 5
23	3, 3	3, 5	1, 9	2, 3	1, 8	1, 9	2, 1	2, 2	1, 1	1, 8	1, 2	2, 2	49, 2	47, 8	49, 4	52, 5	48, 6	46, 4	48, 5	53, 7
24	2, 8	3, 2	2, 4	2, 1	2, 5	2, 1	1, 9	1, 9	1, 3	2, 3	1, 3	1, 9	53, 3	52, 4	53, 2	58, 0	53, 1	51, 4	53, 1	59, 5

Med	3, 7	3, 7	3, 3	3, 2	2, 3	2, 4	2, 3	2, 3	1, 3	2, 4	1, 4	2, 3	48, 5	48, 4	48, 1	47, 4	49, 4	48, 9	49, 0	49, 2
SD	1, 07	0, 96	1, 12	1, 18	0, 43	0, 34	0, 40	0, 33	0, 27	0, 30	0, 29	0, 34	4, 62	3, 98	5, 20	7, 81	3, 05	3, 32	3, 61	4, 41

**Table 5:** Description of radiographic signs associated with the claudication score (CS) and radiographic score (RS) signs.

Animal	CS	Description of radiographic signs	RS
1	0	EOBF and JSC, gas opacity in white line and bone remodeling in apex 3P with hoof rupture	3
2	0	EOBF and JSC, bone remodeling in apex 3P and gas opacity in dorsal lamellae	6
3	0	EOBF and JSC, bone remodeling in apex 3P, gas opacity in abaxial apex side, jagged surface contour in flexor tubercle, and widening of vascular channels	3
4	0	EOBF and JSC, gas opacity in white line with resected hoof wall, concave groove of the dorsal wall of the claw, and widening of vascular channels	3
5	0	EOBF and JSC, bone remodeling in apex 3P, concave groove of the dorsal wall of the claw, widening of vascular channels, and gas opacity in apex	3
6	0	EOBF and JSC, concave groove of the dorsal wall of the claw, gas opacity in white line and apex, and widening of vascular channels	3
7	0	EOBF and JSC, bone remodeling in apex 3P, spiculated periosteal reaction axial e abaxial, concave groove of the dorsal wall of the claw, and fissure in apex 3F	6
8	0	EOBF and JSC, bone remodeling in apex 3P, osteolysis in dorsal wall 3F, concave groove of the dorsal wall of the claw, and widening of vascular channels	4
9	0	EOBF and JSC, widening of vascular channels, bone remodeling in apex 3P, gas opacity in white line, osteophytes in dorsal wall of the claw, and gas opacity in dorsal lamellae	6
10	0	EOBF and JSC, bone remodeling in apex 3P, concave groove of the dorsal wall of the claw, and fissure in 3P	6
11	0	EOBF and JSC, bone remodeling in apex 3P, widening of vascular channels, gas opacity in white line, and gas opacity in dorsal lamellae	6
12	0	EOBF and JSC, bone remodeling in apex 3P, widening of vascular channels, osteophytes in dorsal wall of the claw, and concave groove of the dorsal wall of the claw	2
13	0	EOBF and JSC, bone remodeling in apex 3P and osteolysis in apex 3P	4
14	0	EOBF and JSC, bone remodeling in apex 3P, widening of vascular channels, osteolysis in apex 3P, and jagged surface contour in flexor tubercle	2
15	1	EOBF and JSC, bone remodeling in apex 3P, gas opacity in white line and apex of 3F, and widening of vascular channels	3
16	1	EOBF and JSC, widening of vascular channels, circular bone lysis with sclerosis in axial apex, and osteolysis in apex 3P	4
17	1	EOBF and JSC, osteophytes in axial apex with gas opacity around, and gas opacity in dorsal lamellae	6
18	2	EOBF and JSC, osteolysis in apex 3P, and gas opacity in white line	5
19	2	EOBF, jagged surface contour 3P, osteolysis in apex 3P, and widening of vascular channels	4
20	2	EOBF and JSC, bone remodeling in apex 3P, widening of vascular channels, osteophytes in apex 3P, and lise óssea em apice 3F	4
21	2	EOBF and JSC, bone remodeling in apex 3P, concave groove of the dorsal wall of the claw, osteophytes in apex 3P, and osteolysis in apex 3P	4
22	3	EOBF and JSC, bone remodeling in apex 3P, gas opacity in white line, jagged surface contour in flexor tubercle, widening of vascular channels, concave groove of the dorsal wall of the claw, and osteophytes in apex 3P	3
23	3	EOBF and JSC, bone remodeling in apex 3P, gas opacity in apex, jagged surface contour in flexor tubercle, widening of vascular channels, and concave groove of the dorsal wall of the claw	3
24	3	EOBF and JSC, bone remodeling in apex 3P, osteophytes in apex 3P, osteolysis axial in apex 3P, and gas opacity in dorsal lamellae	6

## Data Availability

All data used to support the findings of this study are included within the article.
